# From large language models to multimodal AI: a scoping review on the potential of generative AI in medicine

**DOI:** 10.1007/s13534-025-00497-1

**Published:** 2025-08-22

**Authors:** Lukas Buess, Matthias Keicher, Nassir Navab, Andreas Maier, Soroosh Tayebi Arasteh

**Affiliations:** 1 Pattern Recognition Lab, Friedrich-Alexander-Universität Erlangen-Nürnberg, Erlangen, Germany; 2 Computer Aided Medical Procedures, Technical University of Munich, Munich, Germany

**Keywords:** Large language models, Generative AI, Multimodal AI, Scoping review

## Abstract

Generative artificial intelligence (AI) models, such as diffusion models and OpenAI’s ChatGPT, are transforming medicine by enhancing diagnostic accuracy and automating clinical workflows. The field has advanced rapidly, evolving from text-only large language models for tasks such as clinical documentation and decision support to multimodal AI systems capable of integrating diverse data modalities, including imaging, text, and structured data, within a single model. The diverse landscape of these technologies, along with rising interest, highlights the need for a comprehensive review of their applications and potential. This scoping review explores the evolution of multimodal AI, highlighting its methods, applications, datasets, and evaluation in clinical settings. Adhering to PRISMA-ScR guidelines, we systematically queried PubMed, IEEE Xplore, and Web of Science, prioritizing recent studies published up to the end of 2024. After rigorous screening, 145 papers were included, revealing key trends and challenges in this dynamic field. Our findings underscore a shift from unimodal to multimodal approaches, driving innovations in diagnostic support, medical report generation, drug discovery, and conversational AI. However, critical challenges remain, including the integration of heterogeneous data types, improving model interpretability, addressing ethical concerns, and validating AI systems in real-world clinical settings. This review summarizes the current state of the art, identifies critical gaps, and provides insights to guide the development of scalable, trustworthy, and clinically impactful multimodal AI solutions in healthcare.

**Supplementary Information:**

The online version contains supplementary material available at 10.1007/s13534-025-00497-1.

## Introduction

Generative artificial intelligence (AI), exemplified by models like ChatGPT, has drawn widespread attention for its ability to process and generate human-like text, substantially advancing various domains. In healthcare, these models have rapidly transformed traditional approaches by offering capabilities beyond conventional data analysis [1, 2]. For instance, large language models (LLMs) have been applied in tasks such as summarizing medical records [3], assisting in diagnostic reasoning [4], and conducting bioinformatics research [5]. These advancements highlight the ability of LLMs to process and interpret complex clinical language, improving efficiency and accuracy across tasks such as radiology reporting. Recent studies further demonstrate their impact, showing that AI-generated draft radiology reports can reduce reporting time by about 25% while maintaining diagnostic accuracy [6], thus addressing workload challenges in clinical practice [7].

**Fig. 1:**
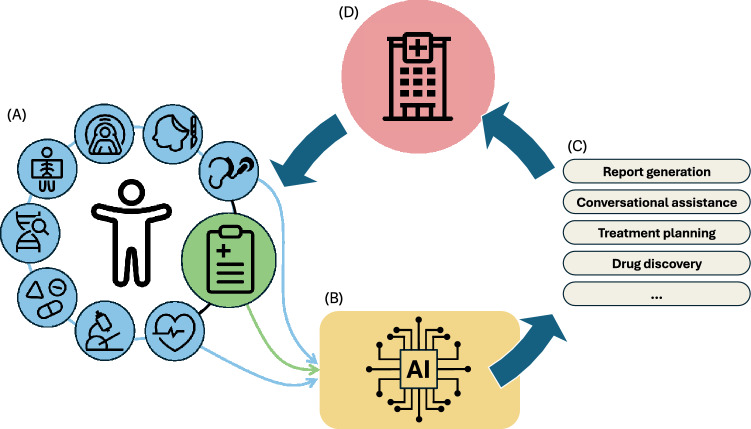
Multimodal AI pipeline in healthcare: **A** Diverse medical data modalities (e.g., images, genomics, and clinical notes) are collected and processed, **B** transformed into unified representations by AI models, **C** used to generate insights such as reports, conversational assistance, and treatment plans, and **D** refined through iterative feedback to continuously optimize data collection and AI performance

However, healthcare data extends far beyond clinical texts, encompassing diverse modalities such as medical images [8, 9], laboratory results [10, 11], and genomic data [12]. To address this diversity, multimodal AI systems have emerged, integrating these data types within a single model to support more comprehensive and clinically relevant decision-making. Recent advancements in this field mark a shift beyond language-focused tasks toward complex, multimodal data integration [13–15]. These systems hold potential for improved diagnostic accuracy and broader applications, from predictive analytics to complex interventional support [16]. Figure 1 illustrates how such models transform heterogeneous medical inputs into clinically meaningful insights through an iterative pipeline.

Several recent review articles have provided valuable overviews of multimodal AI and LLMs. Comprehensive surveys of multimodal large language models (MLLMs) in the broader computer vision domain were presented by Yin et al. [17] and Wang et al. [18], highlighting recent advancements, providing a summary of architectural developments, and identifying key trends in model evolution. A broader perspective on multimodal approaches in healthcare was provided by Kline et al. [19] and Acosta et al. [1]. He et al. [20] present a comprehensive collection of foundation models, spanning from image-only architectures to advanced multimodal models.

While previous reviews provide essential insights, the dynamic and rapidly evolving nature of this field necessitates an up-to-date and focused exploration of recent developments in LLM-based multimodal AI for medicine. This review aims to fill this gap by providing a comprehensive overview of the evolution from text-only LLMs to multimodal AI systems in medicine, with a particular emphasis on recent advancements. Unlike prior reviews, we also discuss evaluation methods specifically tailored to the challenges and requirements of medical generative AI, ensuring real-world clinical utility and reliability.

To guide this review, we formulated the following research questions:

- What methods are commonly used in the development of generative AI for healthcare applications?
- What datasets support the development of generative AI in medical contexts?
- Which evaluation metrics are employed to assess the utility of generative AI models in medical contexts?

In the following sections, we first outline the methodology employed for literature collection and selection, detailing the search strategy, inclusion criteria, and data extraction processes used to ensure a comprehensive review. We then present our findings, emphasizing the shift from text-only LLMs to multimodal AI systems in medicine, with a particular focus on their applications, datasets, model architectures, and evaluation metrics. Our results reveal a significant shift towards multimodal models, which are driving innovation across various areas of healthcare. However, persistent challenges remain, particularly in the evaluation of these models, including the assessment of their reliability, clinical relevance, and generalizability. Finally, we provide an outlook on the future of generative AI in medicine, offering insights to guide further research and development in this rapidly evolving field.

## Methods

Our scoping review followed the Preferred Reporting Items for Systematic Reviews and Meta-Analyses extension for Scoping Reviews (PRISMA-ScR) [21, 22], which provides a standardized framework for methodological transparency in scoping reviews. This section details the data collection methods used in our review. The complete PRISMA-ScR checklist is available in Supplementary Table S.1.

### Eligibility criteria

We included studies published between January 2020 and December 2024 to capture recent advancements in the rapidly advancing field of generative AI in medicine. Only original research in English was eligible, as our focus is on primary contributions rather than synthesized findings. Review and meta-analysis papers were therefore excluded. We included peer-reviewed conference and journal publications, alongside manually selected preprints with high relevance and potential impact. To ensure a comprehensive overview, foundational dataset papers published before 2020 were also included when they were widely used in the selected studies or remained relevant for benchmarking. This approach ensured a focus on current, state-of-the-art developments in multimodal AI applications in medicine.

### Information sources

We performed a systematic search in PubMed, IEEE Xplore, and Web of Science, employing a standardized set of keywords derived from our research objectives. Full search queries are detailed in Supplementary Table S.2. The searches, conducted on October 1, 2024, were imported into Rayyan [23], a web-based tool designed to facilitate literature screening and semi-automated duplicate removal.

### Search strategy

The literature search consisted of a systematic database search structured into two subsearches to capture the development and application of text-only LLMs and multimodal models in medicine. The first subsearch targeted text-only LLMs using the keyword groups "medical" and "language model". The second subsearch focused on multimodal models, using three groups of keywords: "multimodal", "medical" and "language model". The full search queries, including the specific combinations used, are provided in Supplementary Table S.2. Additionally, a manual search was performed to identify recent preprints, datasets, and other resources not captured by the database search, which continued through the end of 2024 to ensure the inclusion of the most current and impactful studies.

### Inclusion and exclusion criteria

The selection process began with structured database queries, followed by duplicate removal, title and abstract screening, and subsequent full-text reviews for potentially relevant papers. We excluded articles that were non-medical or lacked methodological novelty. To ensure balanced representation across application areas, we aimed for proportional inclusion from prevalent fields, such as X-ray report generation.

### Synthesis of results

The selected papers were categorized through a two-step process. First, they were grouped by topics, including text-only LLMs, multimodal models, datasets, and evaluation metrics. Within each topic, papers were further categorized based on their application areas. This dual-layer categorization provides a structured overview of developments in generative AI for medicine, illustrating the progression from text-only LLMs to multimodal models. Key publications are summarized through narrative descriptions and tables, offering insights into methodological approaches, application domains, datasets, and evaluation frameworks to provide a comprehensive understanding of current trends and challenges. Table 1 (text-only LLMs), Table 2 (text-only datasets), Table 3 (contrastive learning methods), Table 4 (MLLMs), Table 5 (multimodal datasets), and Table 6 (evaluation metrics) summarize the results.

## Included studies

**Fig. 2:**
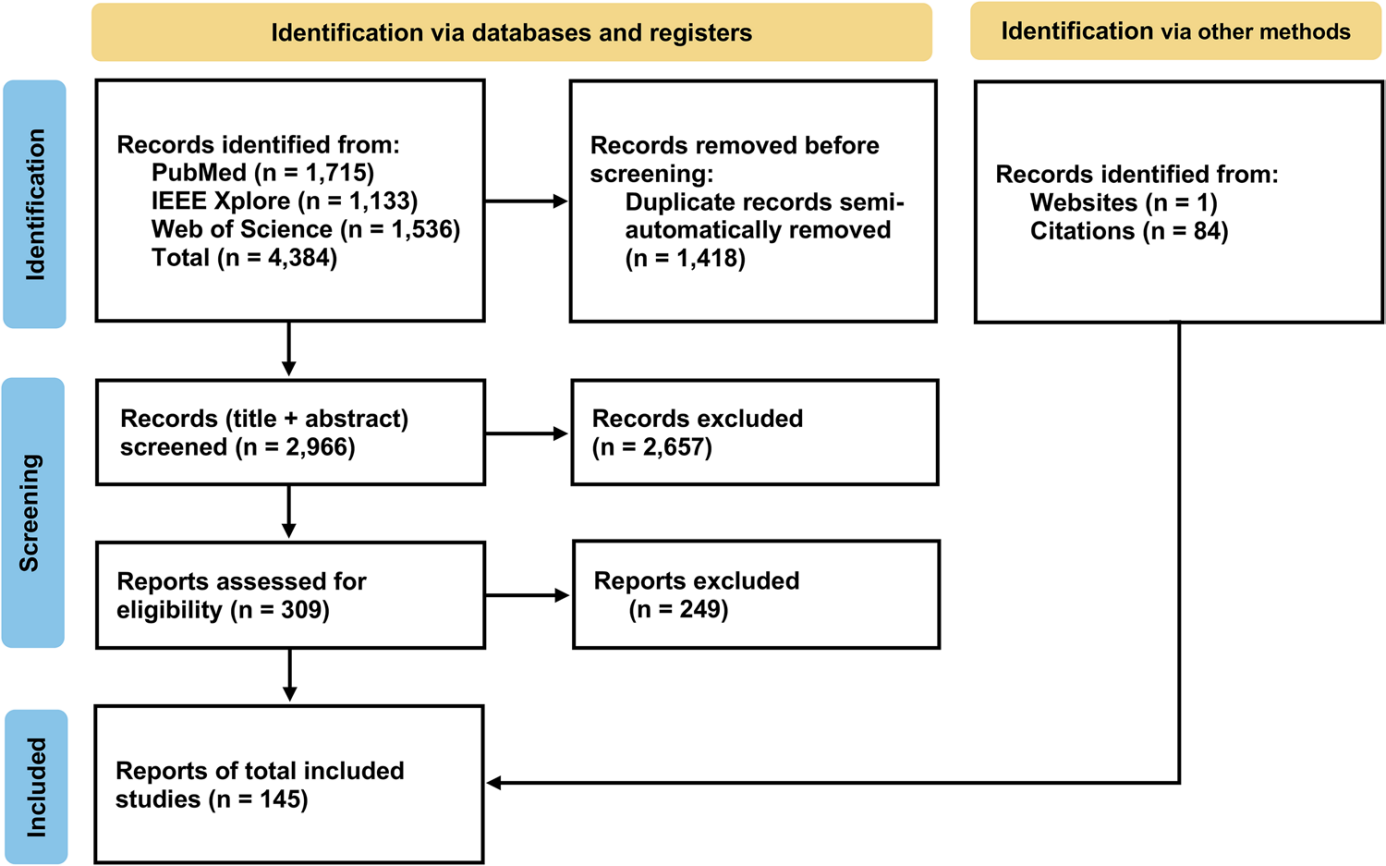
PRISMA flow diagram illustrating the study selection process for the scoping review. The diagram shows the number of records identified through database searches and manual searches, the removal of duplicates, the screening of titles and abstracts, the review of full-text articles, and the final inclusion of studies in the review

A total of 4,384 papers were retrieved from three databases. After removing duplicates, 2,656 articles were excluded during the initial screening based on their titles and abstracts, following the predefined inclusion and exclusion criteria. The remaining articles underwent a full-text review, during which both relevance and topic diversity were considered to avoid overrepresentation of similar studies. This step led to the exclusion of an additional 249 papers. Ultimately, 60 papers from the database search were included in the review. Additionally, 84 papers were identified through manual searches to capture the most current and relevant studies not covered in the database queries. Figure 2 provides an overview of the full screening process. In total, 145 papers were included in this review.

## Language models in medicine

Mono-modal LLMs, which process textual data exclusively, have laid the foundation for the development of multimodal systems, demonstrating remarkable capabilities in understanding and generating human-like text. In the medical domain, LLMs demonstrated high effectiveness in processing and analyzing complex clinical data, enabling advancements in applications such as clinical documentation, medical literature summarization, and diagnostic support [3, 24]. Their success is rooted in the transformer architecture [25], which uses self-attention mechanisms to capture contextual relationships and long-range dependencies in text. Language models use self-supervised learning objectives such as masked language modeling or causal language modeling, which allow them to learn from vast unlabeled datasets. This enables scalable pretraining and contributes to their effectiveness when adapted to medical applications.

### Language model methods

Language models for medical applications (Table 1) differ in their architectures and adaptation strategies. Early pretrained language models (PLMs) such as BioBERT [26] and BioALBERT [27] are based on the BERT architecture and pretrained on biomedical corpora using masked language modeling. They are typically finetuned for specific tasks like named entity recognition (NER) or question answering (QA). These models are optimized for understanding and classification rather than open-ended generation, and thus do not fall under the current definition of modern LLMs.

In contrast, modern LLMs are generally generative and adapted for medical use through instruction-tuned supervised finetuning (SFT), enabling capabilities like zero-shot or few-shot generalization across diverse tasks. Prominent examples include BioMistral [28], ChatDoctor [4], and Med-PaLM [29].

In contrast to SFT, prompt engineering techniques have emerged as a lightweight alternative for guiding pretrained models without additional training, relying on carefully designed input prompts to achieve strong task performance in medical text understanding and generation [30].

Advanced alignment techniques such as reinforcement learning from human feedback (RLHF) have been developed to further refine the outputs of LLMs for medical applications. RLHF leverages reward models trained on expert feedback to align model responses with clinical expectations. However, due to the cost of obtaining expert feedback in the healthcare domain, reinforcement learning from AI feedback (RLAIF) has emerged as an alternative [31]. This technique replaces human feedback with evaluations from auxiliary AI models, reducing reliance on scarce human resources while maintaining alignment capabilities. A notable example is HuatuoGPT [32], which uses RLAIF for clinical alignment.

**Table 1:** Summary of language model methods categorized by application to clinical text and bioinformatics

Study	Downstream task
*Clinical text*	
Almanac [33]	QA
BioALBERT [27]	NER
BioBERT [26]	NER, QA
BioGPT [34]	Classification, QA
BioMistral [28]	QA
ChatDoctor [4]	Dialogue
ChestXRayBERT [3]	Summarization
DRG-LLaMA [35]	Classification
GatorTron [36]	QA
HuatuoGPT [32]	Dialogue
HuatuoGPT-o1 [32]	Dialogue
Johnson et al. [37]	Deidentification
Kresevic et al. [38]	Summarization
Mahendran and McInnes [39]	NER
MAPLEZ [40]	Classification
Med-BERT [41]	NER
MedAlpaca [42]	QA
MEDITRON-70B [43]	QA
MED-PaLM [29]	QA
MMed-Llama 3 [44]	QA
Mu et al. [45]	Classification
NYUTron [24]	Clinical outcome prediction
PMC-LLaMA [46]	QA
PodGPT [47]	QA
RadBERT [48]	Classification, Summarization
Schmidt et al. [49]	Error detection
*Bioinformatics*	
AlphaFold [5]	Structure prediction
BioPhi [50]	Antibody design
CADD v1.7 [51]	Scoring
DNABERT [52]	Structure analysis
Geneformer [53]	Classification
Hie et al. [54]	Antibody design
MSA Transformer [55]	Structure analysis
ProGen [56]	Structure prediction
ProtGPT2 [57]	Protein design
ProtTrans [58]	Structure analysis
scBERT [59]	Classification
ToxinPred 3.0 [60]	Classification

The table includes method names and target applications

NER, named entity recognition; QA, question answering

Another recent development in model adaptation is chain-of-thought (CoT) prompting, a technique where models generate intermediate reasoning steps before producing a final answer. By breaking down complex tasks into substeps, CoT enhances model explainability and task performance, which is especially valuable in the medical domain as it not only improves accuracy but also increases trust in the model’s reasoning process. For example, HuatuoGPT-o1 [61] applies CoT prompting to improve medical response clarity and ensure step-by-step diagnostic reasoning.

An additional adaptation technique is retrieval augmented generation (RAG) [62], which equips LLMs with mechanisms to query external knowledge bases during inference. This approach enables models to access up-to-date information, such as medical guidelines or recent research findings, without requiring retraining. For instance, Almanac [33], ChatDoctor [4], and RadioRAG [63] combine generative capabilities with retrieval systems. However, maintaining the retrieval database and ensuring its comprehensiveness pose ongoing challenges [4, 64].

### Language model applications

LLMs have revolutionized various applications in biomedical language processing, demonstrating utility across a range of tasks. In named entity recognition, they enable the extraction of critical medical entities, such as diseases, drugs, and symptoms from unstructured text. This capability supports clinical data annotation, which is crucial for automated clinical decision support systems [27].

Dialogue systems represent another application of LLMs in medicine. Models like ChatDoctor [4] and HuatuoGPT [32] facilitate patient interactions, simulate doctor-patient consultations, and assist in providing medical information and guidance. These systems aim to reduce barriers to medical access by providing instant responses.

In summarization tasks, medical LLMs condense lengthy electronic health records (EHRs) into concise summaries. This application significantly reduces the documentation burden on healthcare providers and aids decision-making by presenting critical patient information in a structured format [3, 65].

Deidentification and privacy-preserving applications are critical areas where LLMs contribute to medical data management by safeguarding patient confidentiality in sensitive clinical texts. LLMs can automate the removal of protected health information from medical documents by anonymizing identifiers such as names and dates while preserving data utility [37, 40].

Text classification is another important application area for LLMs in medicine. These models have been used to categorize medical literature and to predict patient outcomes based on clinical text, highlighting their ability to extract structured insights from unstructured data [24].

In bioinformatics, LLMs have expanded beyond language processing to analyze biological sequences like DNA, RNA, and proteins. Models such as DNABERT [52] have advanced gene annotation, while AlphaFold [5] has achieved groundbreaking success in protein structure prediction.

### LLM datasets

The development of medical LLMs relies on diverse and specialized datasets that capture the complexity of medical language, context, and tasks. These datasets fall into categories such as clinical text, domain-specific literature, conversational data, and bioinformatics resources, each serving distinct purposes in the development of medical LLMs. These datasets enable general-purpose LLMs to align with the medical domain, which is critical for achieving reliable and accurate outputs in clinical settings.

Clinical text datasets play a central role in training medical LLMs (see Table 2). For instance, EHR datasets like MIMIC-IV [66] provide a rich source of structured and unstructured clinical data, commonly used for tasks such as summarization and NER, which are both essential for automating documentation and decision-making processes in healthcare. The eICU-CRD dataset [67], another EHR resource, focuses on intensive care unit patient data, further broadening the scope of potential applications.

To introduce domain-specific knowledge into LLMs, datasets like GAP-Replay [43] and MedC-K [46], composed of biomedical literature and textbooks, are essential. These datasets are designed to equip models with the specialized terminology and reasoning patterns found in biomedical research and education.

For conversational AI in medicine, dialogue datasets are crucial. MedDialog [68] provides examples of doctor-patient interactions, enabling LLMs to learn medical dialogues, including patient concerns, physician responses, and diagnostic reasoning. These datasets are essential for developing medical conversational assistance systems capable of simulating clinical dialogues and supporting in patient education, diagnostic reasoning, and post-treatment follow-ups.

Bioinformatics datasets extend the scope of LLM applications beyond clinical text, supporting tasks in genomics and molecular biology. Resources like AlphaFold DB [5] and UniProtKB [69] provide structured data for protein structure and sequence analysis, making them valuable for drug discovery and molecular research. Similarly, genomic datasets such as GENCODE [12] and GenBank [70] offer data for tasks like gene prediction, helping models to better understand complex biological patterns.

**Table 2:** Summary of datasets used for training medical LLMs, categorized into clinical text and bioinformatics data

Dataset	Size	Application
*Clinical text*		
eICU-CRD [67]	200K instances	EHR
GAP-Replay [43]	48.1B tokens	Literature
MedDialog-EN [68]	250K dialogues	Dialogue
MedC-K [46]	4.8M papers, 30K textbooks	Literature
MedC-I [46]	202M tokens	Dialogue, QA
Medical Meadow [42]	160K instances	QA
MIMIC-IV [66]	299K patients	EHR
MMedC [44]	25.5B tokens	Multilingual literature
MultiMedQA [29]	213K instances	QA
*Bioinformatics*		
AlphaFold DB [5]	200M entries	Protein Design
CPTAC Data Portal [71]	NA	Genomics, Protein Design
GenBank [70]	NA sequences	Genomics
GENCODE [12]	NA	Genomics
UniProtKB [69]	227M sequences	Protein Design

The table includes dataset names, sizes, and primary application areas

NA, not available; NER, named entity recognition; QA, question answering; EHR, electronic health record

## Multimodal language models in medicine

By showcasing the potential of LLMs in processing clinical text, these models have established a strong foundation for integrating additional modalities, leading to the development of multimodal language models specifically designed for healthcare. Multimodal models combine diverse data types, such as text and medical images, to tackle complex medical tasks, including report generation [72, 73], image-text retrieval [74, 75], and medical consultation [14]. By building on advancements in LLMs, multimodal language models improve the integration and contextual understanding of multimodal medical data. This section provides an overview of recent architectures and methods addressing the unique challenges posed by multimodal medical data.

### Architectures

**Fig. 3:**
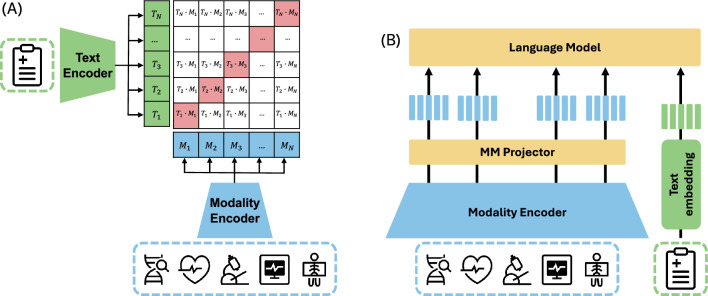
Multimodal architectures: **A** CLIP-based models, which align embeddings of different modalities in a shared latent space for retrieval or classification; and **B** MLLM-based models, which integrate multimodal inputs directly into the language model for generative tasks such as reporting or reasoning

Before presenting the literature, we outline two distinct architectural paradigms in multimodal AI: contrastive models (e.g., CLIP [76]) and generative multimodal large language models (MLLMs) (see Fig. 3). Contrastive models learn joint embeddings of different modality pairs and are primarily used for tasks such as retrieval or classification. While they do not support language generation or multimodal reasoning, they form the foundation for many downstream applications and are often used to pretrain modality encoders for MLLMs. In contrast, MLLMs directly integrate multimodal inputs into a language model to generate natural language outputs and perform complex reasoning.

CLIP [76] is designed to align different modalities, such as image and text, in a shared embedding space. Although originally developed for image-text pairs, its framework can be extended to other modalities, making it a versatile tool for various multimodal learning tasks. By jointly training on paired modalities data, it excels in tasks like zero-shot image classification [74, 77], where new classes can be recognized without additional training. This makes CLIP particularly useful for situations where annotated medical data is limited.

On the other hand, MLLMs, such as LLaVA [78], extend the capabilities of LLMs by integrating non-textual data directly into their embeddings. This integration allows for a more holistic understanding of complex datasets, combining linguistic context with multimodal features like images or clinical measurements. These models excel in tasks such as radiology report generation [72, 73], question answering about medical images [79, 80], and decision support in diagnosis [13, 77, 81].

By leveraging complementary strengths, these architectures address the diverse challenges posed by multimodal medical data. CLIP is effective for aligning different data modalities, while MLLMs excel in diagnostic reasoning, together forming a powerful combination for improving multimodal AI in medicine.

### Contrastive multimodal methods

Contrastive models like CLIP and its medical variants align paired inputs in a shared embedding space and are widely used for representation learning. Table 3 summarizes recent CLIP-based approaches across modalities.

**Table 3:** Summary of multimodal CLIP-based methods

Study	Modalities	Application
BiomedCLIP [82]	Medical images, Descriptions	Classification, Retrieval, Visual QA
BioViL [83]	X-ray, Reports	Classification, Grounding
BioViL-T [84]	X-ray, Reports	Classification, Grounding, Reporting
CheXzero [74]	X-ray, Reports	Classification, Retrieval
ConVIRT [85]	X-ray, Reports	Classification, Retrieval
CPLIP [86]	Histopathology images, Descriptions	Classification
CT-CLIP [14]	CT, Reports, Labels	Classification, Retrieval
CT Foundation [87]	CT, Reports	Classification, Retrieval
CXR-RePaiR [75]	X-ray, Reports	Reporting
ETP [88]	ECG, Reports	Classification
FairCLIP [89]	SLO fundus images, Clinical notes	Classification
FiVE [90]	Histopathology images, Descriptions	Classification
FlexR [91]	X-ray, Reports	Classification
GLoRIA [92]	X-ray, Reports	Classification, Retrieval, Segmentation
KAD [93]	X-ray, Reports	Classification
MaCo [94]	X-ray, Reports	Classification
MCPL [95]	X-ray, Reports	Classification
MedImageInsight [96]	Medical images, Descriptions	Classification, Retrieval, Reporting
Med-MLLM [97]	CT, X-ray, Descriptions	Classification, Reporting
Merlin [77]	CT, EHR, Reports	Classification, Retrieval, Reporting, Segmentation
MedViLL [98]	X-ray, Reports	Classification, Retrieval, Reporting, Visual QA
MoleculeSTM [99]	Molecule structure, Descriptions	Retrieval
MolLM [100]	Molecule structures, Descriptions	Retrieval, Molecule description
PLIP [101]	Histopathology images, Descriptions	Classification, Retrieval
Prov-GigaPath [102]	Histopathology images, Reports	Classification
UniMed-CLIP [103]	Medical images, Captions	Classification
Xplainer [104]	X-ray, Reports	Classification

The table includes method names, the modalities utilized (e.g., text and medical images), and the primary tasks addressed, such as image-text retrieval, report generation, and disease classification

QA, question answering

For instance, BiomedCLIP [82] uses contrastive learning to align medical images with paired reports, achieving state-of-the-art results in retrieval tasks. Building on this framework, CheXzero [74] adapts CLIP for zero-shot classification of X-ray images, while CT-CLIP [14] extends this approach to computed tomography (CT) scans. Similarly, UniMed-CLIP [103] enhances this paradigm by using classification datasets augmented by LLM-generated captions to train a foundation model capable of handling various medical image modalities.

More recent efforts have focused on large-scale pretrained models developed by industry leaders, aiming to generalize across diverse medical imaging tasks. Models like CT Foundation [87] and MedImageInsight [96], accessible via application programming interfaces (APIs), exemplify this trend by offering robust pretrained embeddings that address data scarcity in medical imaging and support downstream applications.

While many CLIP-based methods focus on aligning text with medical images, recent approaches have extended this to other modalities. For example, ETP [88] aligns electrocardiogram (ECG) signals [105, 106] with clinical reports, while MolLM [100] pairs chemical structures with textual descriptions to support drug discovery.

### Multimodal LLM methods

LLM-based methods, in contrast to CLIP approaches, directly integrate multimodal inputs into the language model’s embeddings, enabling more complex reasoning and generative tasks. These approaches rely on modality-specific encoders to process non-textual data, converting them into feature embeddings compatible with the LLM’s text-based representation space (Table 4). For instance, SkinGPT-4 [107] and RaDialog [73] integrate features from two-dimensional (2D) images, while models like Merlin [77] and CT-CHAT [14] extend this capability to volumetric three-dimensional (3D) CT data. Some models, such as MAIRA-2 [72] and AutoRG-Brain [108], further ground text predictions by incorporating bounding boxes and segmentation masks, enabling interactive, region-based report generation for enhanced explainability [109].

Current advancements also focus on text-guided segmentation and synthetic medical image generation. Text-guided segmentation models like LViT create segmentation masks from textual prompts, enabling tasks such as tumor detection and organ identification [110]. Beyond segmentation, synthetic image generation has emerged as another multimodal approach for data augmentation and model training. Methods such as GenerateCT [111] for CT volumes and RoentGen [112] for X-rays use text-conditioned diffusion models to produce realistic medical images [113].

Generalist models, such as BiomedGPT [13] and MedVersa [80], unify multiple modalities and tasks either through shared representations, by combining specialized expert models under a common orchestrator, or by employing mixture-of-experts (MoE) architectures with learnable routing mechanisms [114]. These models employ specialized modules to process different modalities while a central language model coordinates their outputs, enabling tasks such as classification, segmentation, retrieval, and visual QA. This approach highlights the scalability and versatility of generalist models in addressing complex multimodal challenges in medicine.

**Table 4:** Summary of multimodal MLLM-based methods

Study	Modalities	Downstream task
Alsharid et al. [115]	US video, Transcriptions, Gaze data	Captioning
AutoRG-Brain [108]	MRI, Reports, Masks	Reporting, Grounding
BiomedGPT [13]	Medical images, Literature, EHR	Reporting, Summarization, Visual QA
BioMed-VITAL [116]	Medical images, Instructions	Visual QA
ChatCAD [117]	X-ray, Reports	Reporting
CheXagent [118]	X-ray, Reports	Classification, Reporting, Grounding
COMG [119]	X-ray, Reports, Masks	Reporting
CT-CHAT [14]	CT, Reports	Reporting, Visual QA
FFA-GPT [120]	Fundus fluorescein angiography, Reports	Reporting, Visual QA
GenerateCT [111]	CT, Reports	Image generation
Huh et al. [121]	X-ray, Reports	Reporting
LLaVA-Med [15]	Medical images, Captions	Visual QA
LViT [110]	CT, X-ray, Masks, Text annotations	Segmentation
M3D-LaMed [122]	CT, Reports, Masks	Reporting, Visual QA, Segmentation
MAIRA-2 [72]	X-ray, Reports, Masks	Reporting, Grounding
MAIRA-Seg [123]	X-ray, Reports, Masks	Reporting
Med-Flamingo [124]	Medical images, Captions	Visual QA
Med-MoE [114]	Medical images, Captions	Visual QA
Med-PaLM M [79]	Medical images, Reports, Genomics	Classification, Reporting, Visual QA, Summarization
MedVersa [80]	CT, X-ray, Dermatology images, Reports	Classification, Reporting, Visual QA, Segmentation
MMBERT [125]	Radiology images, Captions	Visual QA
MVG [126]	Medical images, Text	Disease simulation
ORacle [16]	Multi-view images, SSG, Descriptions	OR scene graph prediction
PathChat [127]	Histopathology images, QA-pairs	Visual QA
PathLDM [128]	Histopathology images, Reports	Image generation
QUILT-LLaVA [129]	Histopathology images, QA-pairs	Visual QA
R2GenGPT [130]	X-ray, Reports	Reporting
RaDialog [73]	X-ray, Reports	Reporting, Dialogue
RadFM [81]	Medical images, Reports, Descriptions	Reporting, Visual QA
ReXplain [131]	Video, Reports, Masks	Video report generation
RGRG [109]	X-ray, Reports, Bounding-boxes	Reporting
RoentGen [112]	X-ray, Reports	Image generation
SkinGPT-4 [107]	Dermatology images, Clinical notes	Visual QA, Dialogue
Surgical-VQLA++ [132]	Surgical images, QA-pairs	Visual QA
Universal Model [133]	CT, Masks, Descriptions	Segmentation
Vote-MI [134]	MRI, Reports	Visual QA

The table includes method names, the modalities utilized (e.g., text and medical images), and the primary tasks addressed, such as report generation, visual QA, and disease classification

QA, question answering

### Multimodal LLM applications

MLLMs have been increasingly applied across diverse medical tasks, showcasing their potential to transform clinical workflows and decision support systems. This section highlights key applications where MLLMs contribute to improving healthcare.

A key advancement in multimodal AI is generalist models capable of handling diverse medical data types and tasks. Models such as BiomedGPT [13] and RadFM [81] support a wide range of imaging modalities and anatomical regions, enabling comprehensive diagnostic assistance across multiple specialties.

Radiology report generation remains one of the most important applications of MLLMs in healthcare, providing detailed textual descriptions directly from medical images. Systems such as MAIRA-2 [72] and RaDialog [73] have demonstrated their ability to generate comprehensive reports from X-rays, while CT-CHAT [14] and AutoRG-Brain [108] extend this capability to CT and magnetic resonance imaging (MRI) scans, respectively. These tools assist radiologists by automating preliminary reporting and standardizing documentation, potentially reducing reporting delays.

Visual QA systems support clinicians in querying medical images using natural language prompts, supporting real-time decision-making and diagnostic interpretation. For instance, models like LLaVA-Med [15] and Med-Flamingo [124] provide concise, contextually relevant answers to clinical queries, assisting radiologists and physicians in complex cases.

Synthetic medical image generation has become increasingly important for data augmentation and simulating rare pathological conditions. Models like GenerateCT [111] and RoentGen [112] generate realistic CT and X-ray images from textual prompts, enhancing dataset diversity.

Semantic scene modeling is another emerging application where models create structured representations of complex environments, such as the operating room. For example, ORacle [16] generates semantic scene graphs to assist with surgical planning and intraoperative navigation by representing tools, anatomy, and procedural stages in a comprehensive framework.

Finally, systems like ReXplain [131] aim to bridge communication gaps between clinicians and patients. By transforming radiology reports into patient-friendly video summaries, these models provide an accessible way to convey complex clinical information, further highlighting multimodal AI’s potential to improve patient care.

### Multimodal LLM datasets

Multimodal datasets integrating images, text, and other clinical information (Table 5) are essential for tasks such as radiology report generation, visual QA, and cross-modal retrieval. These datasets not only enable effective model training but are also crucial for ensuring fairness and generalization in medical AI systems. A range of multimodal datasets has been curated to support various medical imaging and diagnostic tasks.

**Table 5:** Summary of multimodal datasets used for medical AI, grouped by modality categories

Dataset	Modalities	Size	Application
*2D-image-text*			
CheXpert [135]	X-ray, Reports, Labels	224K triplets	Chest X-ray
CheXinstruct [118]	X-ray, Instructions	8.5M instruction triplets	Chest X-ray
Harvard-FairVLMed [89]	SLO fundus images, Demographics, Notes	10K samples	Ophthalmology
MedTrinity-25 M [136]	Medical images, Captions, Bounding-boxes	25M pairs	Medical imaging
MedVidQA [137]	Medical videos, Labels, QA-pairs	6K videos, 6K labels, 3K QA	Medical videos
MIMIC-CXR [8]	X-ray, Reports	377K images, 227K reports	Chest X-ray
MS-CXR [83]	X-ray, Descriptions, Bounding-boxes	1K image-sentence pairs, Bounding-boxes	Chest X-ray
OmniMedVQA [138]	Medical images, QA	118K images, 127K QA-pairs	Medical imaging
OpenPath [101]	Histopathology images, Captions	208K pairs	Digital pathology
PadChest [139]	X-ray, Reports	160K images, 109K texts	Chest X-ray
PathVQA [140]	Medical images, QA	5K images, 33K QA	Medical imaging
PMC-15 M [82]	Medical images, Captions	15M image-text pairs	Medical imaging
PubMedVision [141]	Medical images, QA	1.3M QA pairs	Medical imaging
Quilt-1 M [142]	Histopathology images, Captions	1M pairs	Digital pathology
Rad-ReStruct [143]	X-ray, Structured reports	3720 images, 3597 Reports	Chest X-ray
SLAKE [144]	Medical images, QA	642 images, 14K QA pairs	Medical imaging
UniMed [103]	Medical images, Captions	5.3M image-text pairs	Medical imaging
VQA-RAD [145]	Radiology images, Captions	315 images, 3.5K QA pairs	Radiology
*3D-volume-text*			
AMOS-MM [146, 147]	CT, Reports, QA	2K image-report pairs, 7K QA	Chest, abdomen, pelvis CT
BrainMD [134]	MRI, Reports, EHR	2.5K cases	Brain MRI
BIMCV-R [148]	CT, Reports	8K image-report pairs	CT
CT-RATE [14]	CT, Reports, Labels	25K triplets	Chest CT
INSPECT [9]	CT, Reports, EHR, labels	23K image-report pairs, EHRs	Chest CT
M3D-Data [122]	CT, Captions, QA, Masks	120K images, 42K captions, 509K QA, 149K masks	CT
RadGenome-Brain MRI [108]	MRI, Reports, Masks	3.4K image-region-report triplets	Brain MRI
RadGenome-Chest CT [149]	CT, Reports, Masks, QA	25K image-report pairs, 665K masks, 1.3M QA	Chest CT
*Others*			
Duke Breast Cancer MRI [150]	Genomic, MRI images, Clinical data	922 subjects	Breast cancer
PTB-XL [151]	ECG signals, Reports, Labels	21K triplets	ECG
PubChemSTM [99]	Chemical structures, Descriptions	280K chemical structure–text pairs	Drug design
SwissProtCLAP [152]	Protein Sequence, Text	441K sequence-text pairs	Protein design

The table lists dataset names, the types of modalities (e.g., text and medical images), dataset sizes, and key applications such as image-text retrieval, report generation, and disease classification

QA, question answering

A substantial proportion of multimodal datasets focus on pairing vision and text data, as this combination is central to tasks where both visual context and descriptive language are critical for diagnostic interpretation. Notable public datasets like MIMIC-CXR [8] and CheXpert [135] provide rich resources for training 2D vision-language models in radiology. These datasets include not only radiology reports but also disease-specific labels, enabling more comprehensive evaluations. For benchmarking report generation, ReXGradient [153], a private benchmark dataset of 10,000 studies collected across 67 medical sites in the United States, offers diverse coverage and serves as a reliable standard for radiology-specific performance evaluation.

Expanding beyond radiology, datasets like Quilt-1 M [142] have introduced multimodal resources covering additional domains such as digital pathology [127, 154].

Recent advancements have also led to datasets tailored for 3D imaging modalities such as CT [9, 14, 146, 148] and MRI [108]. Notably, RadMD [81] integrates both 2D and 3D imaging modalities, supporting a broader range of applications.

In addition to image-text pairs, a few datasets now include task-specific annotations to support specialized applications. For instance, RadGenome-Brain MRI [108] and RadGenome-Chest CT [149] provide segmentation masks, while datasets like MedTrinity-25 M [136] offer bounding box annotations. These annotations are critical for grounding text predictions to specific regions of interest, enhancing both explainability and diagnostic accuracy in multimodal models.

The data formats of multimodal datasets also vary significantly based on their intended use cases. While datasets like OpenPath [101] present images from publicly available sources in formats such as JPEG, datasets like MIMIC-CXR [8] and CT-RATE [14] preserve clinical formats such as Digital Imaging and Communications in Medicine (DICOM) and Neuroimaging Informatics Technology Initiative (NIfTI). These formats are essential for maintaining complete clinical information and enabling compatibility with healthcare systems.

Beyond traditional imaging and text combinations, datasets have also begun exploring additional modalities for specialized biomedical tasks. For example, SwissProtCLAP [152] integrates protein sequence data to support protein design frameworks, highlighting the potential of multimodal datasets to extend AI applications beyond diagnostic imaging into molecular and genomic research.

## Evaluation metrics for generative AI in medicine

Evaluating generative AI in medicine is essential to ensure models produce accurate, clinically relevant, and reliable outputs [155]. This section explores evaluation metrics for both text generation, such as radiology report generation, and image generation, emphasizing the importance of clinical validity and utility. As general-purpose metrics often fall short in capturing medical accuracy, domain-specific approaches are required.

**Fig. 4:**
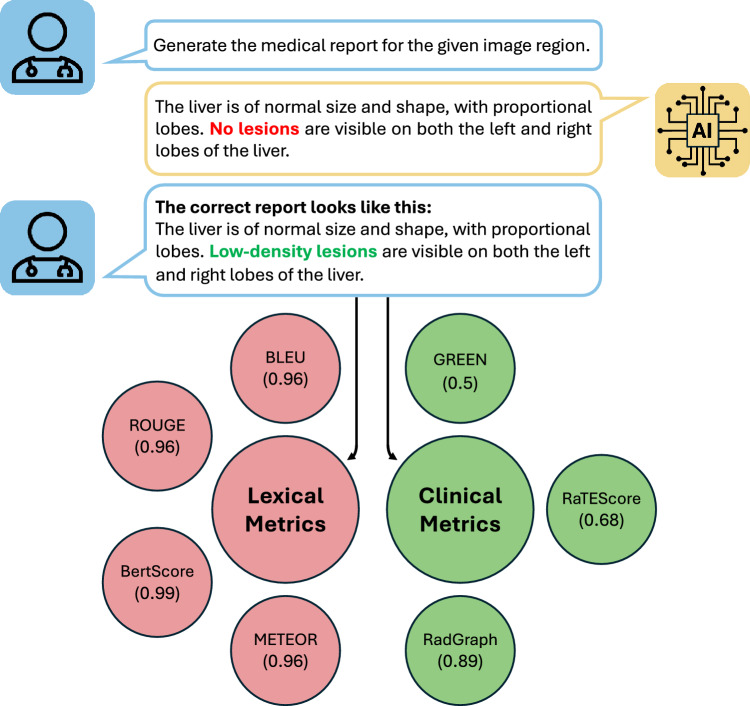
Evaluation of generative AI in medicine: Lexical metrics from the general domain cannot completely capture the clinical correctness as they mainly cover text similarity. Clinically-relevant metrics like GREEN [155], RaTEScore [156], or RadGraph [157] also evaluate the clinical correctness

As report generation is a key application of generative AI in medicine, research has focused on developing robust evaluation strategies. While standard lexical metrics such as BLEU [158], ROUGE [159], and METEOR [160] are commonly used, they often fail to reflect clinical accuracy, as high scores can be achieved despite factually incorrect outputs. These metrics focus on surface-level similarity between generated and reference texts, employing n-gram precision (BLEU), recall of overlapping segments (ROUGE), and flexible word-level matching that accounts for stemming and synonyms (METEOR). However, they lack sensitivity to clinical nuances such as negation, anatomical detail, and factual correctness (see Fig. 4).

To address these shortcomings, several specialized metrics have been proposed for evaluating medical reports (Table 6). RaTEScore [156] is an entity-aware metric that evaluates the overlap of extracted clinical concepts, including anatomical structures, findings, and their relationships. It captures clinically significant variations in expression, such as synonymous terms and negated findings, providing a more informative measure of clinical relevance.

FineRadScore [161] evaluates radiology reports by prompting powerful language models like GPT-4 to assess clinical aspects such as factuality, temporal consistency, and severity. While effective, it depends on access to strong language models, which may limit reproducibility and practical use.

GREEN [155] addresses this limitation by distilling the evaluation capability of large models into a smaller, instruction-tuned language model. It detects and categorizes inconsistencies between generated and reference reports and produces human-interpretable feedback alongside a numerical score. This makes GREEN a practical and clinically grounded tool for both benchmarking and error analysis.

RadFact [72] uses a LLM to evaluate sentence-level factual consistency by comparing generated text to reference reports. In grounding tasks, it also incorporates image annotations to assess whether model predictions are supported by visual evidence.

In addition to text-based evaluation, clinical efficacy can also be assessed using standard classification metrics such as precision, recall, specificity, and F1-score. This is particularly relevant when using label-based datasets [8, 14], where a classifier is used to assign diagnostic labels to generated reports (e.g. CheXbert [162]), allowing comparison to ground truth annotations.

**Table 6:** Evaluation metrics for medical report generation

Metric	Type	Application
CheXbert [162]	Classification	Chest X-ray report labeling
CRAFT-MD [163]	Generative	Conversation evaluation
FineRadScore [161]	Generative	Report evaluation
GREEN [155]	Generative	Report evaluation
Ong Ly et al. [164]	Calibration	Model generalization
RadCliQ [157]	Composite metric	Report evaluation
RadFact [72]	Grounding	Grounded report evaluation
RadGraph-F1 [157]	NER similarity	Report evaluation
RaTEScore [156]	NER similarity	Report evaluation

This table summarizes key metrics used to evaluate generative AI systems in medical report generation, categorized by type and primary application

NER, named entity recognition

Evaluating image generation in medical AI requires considerations beyond standard image quality metrics like Fréchet Inception Distance [165] and mean squared error. Since synthetic medical images are often used for data augmentation or diagnostic training, their clinical utility must be assessed alongside visual quality. One effective strategy involves generating condition-specific medical images and training a classifier on the synthetic data to evaluate its generalization performance on real clinical cases [111]. This ensures that the generated images are not only visually realistic but also contribute to model performance on downstream tasks, such as disease classification and segmentation.

Despite advancements in specialized evaluation metrics for both text and image generation, challenges remain regarding their generalizability across clinical sites and datasets. Frameworks like ReXamine-Global [166] address this by evaluating the robustness of metrics across diverse institutions and data distributions. For text generation, a combination of lexical metrics and clinically grounded assessments is essential to ensure factual correctness and clinical relevance. Similarly, for image generation, both visual quality and downstream clinical utility, such as diagnostic performance on real clinical cases, should be jointly evaluated. Ultimately, a multi-dimensional evaluation approach that considers both data diversity and task-specific requirements is crucial for the safe and effective deployment of generative AI in healthcare.

## Discussion

In this scoping review, we systematically explored the evolution of generative AI in medicine, focusing on LLMs, multimodal LLMs, and their evaluation metrics. Using the PRISMA-ScR framework [21], we collected 145 papers published between January 2020 and December 2024 from PubMed, IEEE Xplore, and Web of Science, complemented by a manual search to ensure comprehensive coverage. Our findings highlight the shift from unimodal LLMs focused on textual tasks to more complex multimodal systems capable of integrating medical images, clinical notes, and structured data. These models have shown promise in enhancing diagnostic support, automating clinical workflows, and reducing the workload of healthcare professionals.

LLMs have advanced biomedical language processing, improving tasks like medical report summarization, named entity recognition, and conversational AI. Adaptation techniques such as supervised finetuning, reinforcement learning, and RAG have further specialized language models for clinical tasks. However, reliance on static datasets like MIMIC-IV [66] limits the ability to capture evolving medical knowledge. Moreover, privacy issues persist due to the need for extensive data deidentification, and dataset biases can affect fairness by overrepresenting specific populations [167, 168].

Multimodal LLMs extend LLM capabilities by integrating multiple data types, such as medical images and text, to address tasks like report generation, cross-modal retrieval, and clinical question answering. Despite these advancements, data heterogeneity remains a challenge, as clinical datasets often vary significantly in format, quality, and completeness across institutions. Additionally, most widely used datasets, such as MIMIC-CXR and CT-RATE [8, 14], focus heavily on radiology, limiting the generalizability of models to other medical domains.

Evaluating generative AI models in medicine requires specialized metrics that go beyond standard language evaluation metrics. While lexical metrics like BLEU [158] and ROUGE [159] are commonly used, they often fail to capture clinical relevance and factual accuracy. To address this, domain-specific metrics such as RadGraph [157], RaTEScore [156], and GREEN [155] have been developed to assess the clinical validity of generated medical reports. However, challenges remain in standardizing evaluation practices across diverse medical tasks and datasets. Most evaluations are limited to radiology, with less attention given to other specialties. The limited availability of well-annotated multimodal datasets with fine-grained clinical labels further complicates performance benchmarking. Additionally, only a few benchmarking frameworks, such as ReXrank [153], offer the ability to neutrally evaluate models on non-public datasets, limiting comparative performance assessments across different models and data sources. Expanding such benchmarks and ensuring their applicability to a broader range of clinical tasks is essential for developing trustworthy generative models in medicine.

While this scoping review provides a comprehensive overview of generative AI advancements in medicine, it has certain limitations. Despite the systematic search strategy using the PRISMA-ScR framework, the literature search may not have captured all relevant studies due to the rapidly evolving nature of the field. To mitigate this, a manual search was conducted alongside the database queries to ensure the inclusion of recent and high-impact publications. Moreover, while efforts were made to cover multiple clinical specialties, there remains an overrepresentation of radiology-focused datasets and models, reflecting a broader trend in the literature. We aimed to balance the inclusion of topics and application areas by diversifying the datasets and models included in our analysis, but certain domains such as pathology and genomics remain less represented due to the current availability of multimodal datasets in these fields.

To further advance the development and responsible deployment of generative AI in medicine, several areas need attention [169–171]. First, evaluation frameworks need to evolve beyond lexical metrics by incorporating clinically grounded assessments and domain-specific error analysis. Second, expanding the diversity of training datasets is critical. The current overrepresentation of western institutions and radiology-focused datasets risks introducing biases that limit global applicability [8, 135]. Future datasets should encompass a wider range of medical specialties, imaging modalities, and patient demographics, with careful attention to privacy protection and data fairness. Third, improving model explainability remains a priority [172, 173]. Techniques such as region-specific grounding can help build clinician trust. Finally, the emergence of generalist models [13, 80] capable of handling multiple modalities and tasks within a unified architecture represents an important step forward, but broader coverage across medical specialties and improved datasets remain essential for widespread adoption.

This scoping review provides a structured analysis of the evolution from unimodal LLMs to multimodal generative AI models in medicine, highlighting their potential for improving diagnostic support, clinical documentation, and decision-making. However, challenges related to data diversity, clinical relevance, model interpretability, and the standardization of evaluation metrics remain critical barriers to widespread adoption. Addressing these challenges through interdisciplinary collaboration, improved datasets, and clinically grounded evaluation strategies will be essential to ensure the responsible deployment of generative AI in healthcare.

## Supplementary Information

Below is the link to the electronic supplementary material.

Supplementary file 1 (pdf 109 KB)

## Abbreviations

AI: Artificial intelligence
API: Application programming interface
CLIP: Contrastive language-image pretraining
CoT: Chain-of-thought
CT: Computed tomography
DICOM: Digital imaging and communications in medicine
ECG: Electrocardiogram
EHR: Electronic health record
LLM: Large language model
MLLM: Multimodal large language models
MRI: Magnetic resonance imaging
NER: Named entity recognition
NIfTI: Neuroimaging informatics technology initiative
PLM: Pretrained language model
PRISMA: Preferred reporting items for systematic reviews and meta-analyses
PRISMA-ScR: Preferred reporting items for systematic reviews and meta-analyses extension for scoping reviews
QA: Question answering
RAG: Retrieval augmented generation
RLHF: Reinforcement learning from human feedback
RLAIF: Reinforcement learning from AI feedback
SFT: Supervised finetuning
